# Habitual glucosamine use, APOE genotypes, and risk of incident cause-specific dementia in the older population

**DOI:** 10.1186/s13195-023-01295-6

**Published:** 2023-09-09

**Authors:** Chun Zhou, Yanjun Zhang, Sisi Yang, Ziliang Ye, Qimeng Wu, Mengyi Liu, Panpan He, Yuanyuan Zhang, Xianhui Qin

**Affiliations:** National Clinical Research Center for Kidney Disease, State Key Laboratory of Organ Failure Research, Guangdong Provincial Institute of Nephrology, Guangdong Provincial Key Laboratory of Renal Failure Research, Division of Nephrology, Nanfang Hospital, Southern Medical University, Guangzhou, 510515 China

**Keywords:** Glucosamine supplement, Vascular dementia, Alzheimer’s disease, APOE genotypes

## Abstract

**Background:**

The relationship of glucosamine use with incident dementia in the older population remains uncertain. We aimed to evaluate the longitudinal association between habitual glucosamine supplement and the risk of cause-specific dementia and examine the possible effect modifiers on this association.

**Methods:**

The study included 214,945 participants over the age of 60 who had available information on glucosamine use and did not have dementia at baseline in the UK Biobank. The APOE genotypes were determined by a combination variant of rs429358 and rs7412. The primary outcome was incident vascular dementia, incident Alzheimer’s disease, and incident frontotemporal dementia, respectively.

**Results:**

Over a median follow-up duration of 12 years, 1039, 1774, and 122 participants developed vascular dementia, Alzheimer’s disease, and frontotemporal dementia, respectively. Overall, habitual glucosamine use was significantly associated with a lower risk of incident vascular dementia (adjusted HR, 0.82; 95%CI, 0.70–0.96), but not significantly associated with incident Alzheimer’s disease (adjusted HR, 1.02; 95%CI, 0.92–1.14) and incident frontotemporal dementia (adjusted HR, 0.95; 95%CI, 0.63–1.43). Moreover, the inverse association between habitual glucosamine use and incident vascular dementia was more pronounced in participants with concomitant supplement of calcium (*P*-interaction = 0.011), and those without concomitant supplement of zinc (*P*-interaction = 0.018). However, APOE ε4 dosage and baseline cognitive function did not significantly modify the relationships of glucosamine use with incident vascular dementia or Alzheimer’s disease (All *P*-interactions > 0.05).

**Conclusions:**

Regardless of APOE genotypes and baseline cognitive function, habitual glucosamine use was significantly inversely associated with incident vascular dementia in the older population.

**Supplementary Information:**

The online version contains supplementary material available at 10.1186/s13195-023-01295-6.

## Introduction

Glucosamine is sold as a prescription drug in most European countries. In other countries, including the USA, Australia, and the UK, approximately 20% of the population may choose to take glucosamine supplements instead of being prescribed by a healthcare professional [1–3].

In addition to the possible symptomatic benefits of glucosamine use on painful osteoarthritis [4–8], recent evidence suggests that glucosamine may modulate inflammation status [9–11], and may therefore be associated with improvements in a range of chronic metabolic disorders, particularly obesity, type 2 diabetes, and cardiovascular disease (CVD) [12, 13]. Furthermore, studies in animal models have linked the use of glucosamine with better cognitive function [14, 15]. A previous cross-sectional study in the UK also found that participants with glucosamine supplementation showed better cognitive performance [16]. Since inflammation, CVD, and impaired cognitive function are all closely related to dementia, especially vascular dementia, we speculate that glucosamine use may be associated with the reduction of dementia, especially vascular dementia. However, only a few studies [17, 18] have investigated the longitudinal association of habitual glucosamine use with incident dementia in the general population and have reported inconsistent results. In addition, despite older adults are at high risk for dementia, no studies have specifically examined the association of glucosamine use with dementia risk in older adults and explored the possible effect modifies on this association, particularly nutrients such as fish oil, minerals, and vitamins that are commonly used in older adults. More importantly, although Apolipoprotein E (APOE) gene polymorphic alleles are a major determinant for Alzheimer disease and play an important role in the risk of vascular dementia [19, 20], whether the APOE genotypes may modify the association glucosamine use and dementia risk has not been examined in the older population. At the same time, none of the previous studies have evaluated whether baseline cognitive function may modify the association between glucosamine use and the risk of dementia.

To address the aforementioned knowledge gaps, we aimed to evaluate the longitudinal association between habitual glucosamine supplement and the risk of cause-specific dementia, especially vascular dementia, and examine the possible effect modifiers on this association.

## Materials and methods

### Study population

The UK biobank is a large-scale, long-term prospective health research study designed to provide in-depth information on the effects of comprehensive exposures on a wide range of health conditions to further promote human health. The UK Biobank included 500,000 residents in the UK, aged between 40 and 69 years at the time of recruitment from 2006 to 2010. Participants were asked to complete a touch-screen questionnaire, a face-to-face nurse interview, and a series of physical measurements, as well as to provide biological samples for genotype and biomarker analysis. Details of the study design have been described in the official website (https://www.ukbiobank.ac.uk/) and previous studies [21]. The study protocol conforms to the ethical standards of the responsible committee on human experimentation (institutional and national) and with the Helsinki Declaration of 1975, as revised in 2008, as reflected in the approval by the North West Multi-Center Research Ethics Committee (06/MRE08/65), and all participants were informed and gave written informed consent prior to the study.

Of the 502,461 participants in the UK Biobank, a total of 214,945 participants were included in the final analysis after excluding participants who withdrew data (*N* = 47), had missing data on glucosamine use (*N* = 6194), reported dementia at baseline (*N* = 587), or were younger than 60 years of age at recruitment (*N* = 280,688) (Supplementary Fig. 1).

### Exposure assessment

The information on habitual glucosamine use was collected from a touchscreen questionnaire. Each participant was asked, “Do you regularly take any of the following supplements?”, and then could select more than one answer from a list of dietary supplements. Habitual glucosamine use was defined as: 1 = yes, 0 = no.

### Covariates assessment

The APOE genotypes were determined by a combination variant of rs429358 and rs7412 [22]. Based on the number of APOE Ɛ4 allele, participants were further divided into the high-risk group (APOE ε4 dosage = 2, ε4/ε4), medium-risk group (APOE ε4 dosage = 1, ε3/ε4), and low-risk group (APOE ε4 dosage = 0, ε2/ε2, ε2/ε3, ε3/ε3) [23] in this analysis.

Information on demographic, lifestyle factors drug use and nutrient supplementation was collected through a touchscreen questionnaire at baseline. The following covariates were included: age, sex, ethnicity, body mass index (BMI), education levels, household incomes, Townsend deprivation index, smoking status, alcohol consumption, physical activity, family history of dementia, self-reported diseases (diabetes, hypertension, arthritis, CVD), and use of drugs (antihypertensive drugs, lipid-lowering drugs, aspirin, non-aspirin non-steroidal anti-inflammatory drug [NSAID], insulin), dietary intakes (cereal, fish, fruit, red meat, vegetables), vitamin supplements (vitamin A, vitamin B, vitamin C, vitamin D, vitamin E, and folic acid), mineral and other supplements (calcium, selenium, iron, zinc and fish oil), and healthy diet scores. BMI was calculated by dividing weight (kg) by the square of standing height (m) for each participant. Optimal physical activity was defined as more than 4 days of vigorous/moderate physical activity in a typical week [24]. The healthy diet score consisted of following dietary goals for ideal cardiovascular health [25, 26]: fruit intake ≥ 3 servings/day, vegetable intake ≥ 3 servings/day, whole grain intake ≥ 3 servings/day, fish intake ≥ 2 servings/day, dairy intake ≥ 2 servings/day, vegetable oil ≥ 2 servings/day, refined grain intake ≤ 2 servings/day, processed meat ≤ 1 serving/day, unprocessed meat ≤ 2 servings/day, and sugar-sweetened beverages intake = 0. If participants achieved one of 10 dietary goals, they will get one point. The range of healthy diet score is 0–10.

The cognitive tests were developed specifically for UK Biobank to enable large-scale computerized administration without staff involvement and are therefore non-standardized. The UK Biobank cognitive tests show a range of validity coefficients that coexist with well-validated standard tests of cognitive ability, and most of the tests tend to have moderate to good retest reliability [27]. Baseline cognitive function tests include verbal and numerical reasoning (‘Reasoning’), processing speed (‘Reaction Time’), attention/working memory (‘Numeric Memory”), visuospatial memory (‘Pairs Matching’), and prospective memory (‘Prospective Memory’) [28]. Except that prospective Memory is a binary variable, the raw scores for all tests were converted to *z*-scores for easy interpretation, and standardized within 5-year age bands [29]. Therefore, the average score is approximately zero and the standard deviation is approximately 1. The signs of the *z*-scores for Reaction Time and Pairs Matching were reversed, so a higher *z*-score for each cognitive function test represents a better performance. In addition, a composite measure of global cognitive function was then calculated by averaging the available z-scores for each participant [30].

### Ascertainment of study outcomes

The study outcome was incident vascular dementia, incident Alzheimer’s disease, and incident frontotemporal dementia, respectively.

Incident vascular dementia, Alzheimer’s disease, and frontotemporal dementia were ascertained from the International Classification of Disease version 10 (ICD-10), ICD-9 coding system. Vascular dementia was defined as ICD-9 codes 290.4, ICD-10 codes F01 and I67.3; Alzheimer’s disease was defined as ICD-9 codes 331.0, ICD-10 codes F00 and G30; frontotemporal dementia was defined as ICD-9 codes 331.1, ICD-10 codes F02.0 and G31.0. The accuracy of dementia ascertainment has been validated previously [31, 32].

The follow-up for each participant was calculated from the date of the first assessment until the first diagnosis date of study outcome, date of death, date of loss to follow-up, or the end of follow-up, whichever came first (February 28, 2018, for Wales, March 31, 2021, for England and Scotland).

### Statistical analysis

Baseline characteristics of study participants were summarized and stratified by glucosamine use status at baseline. Continuous and categorical variables were presented as means (standard deviation: SD) and numbers (proportion), respectively. Differences between groups were determined by *t* tests and chi-square tests, accordingly.

Cox proportional hazard models were applied to calculate the hazard ratios (HRs) and 95% confidence intervals (95%CIs) for associations of glucosamine use with incident risk of vascular dementia, Alzheimer’s disease, and frontotemporal dementia, respectively. The proportional hazards assumption was checked using Schoenfeld residuals, and no violation of this assumption was detected. Basic model was adjusted for age and sex (female or male). Model 1 included the adjustments for age, sex (female or male), ethnicity (white, others), centers, BMI, household incomes (< 18,000, 18,000–30,999, 31,000–51,999, 52,000–10,000, > 100,000 £/year), Townsend deprivation index, smoking status (never, former, current), alcohol consumption (daily or almost daily, 3–4 times a week, once or twice a week, 1–3 times a month, never or special occasions only), optimal physical activity (yes or no), family history of dementia (yes or no), APOE ε4 dosage (0,1,2), self-reported diabetes (yes or no), self-reported hypertension (yes or no), self-reported arthritis (yes or no), history of cardiovascular disease (yes or no), antihypertensive drugs (yes or no), lipid treatment (yes or no), aspirin use (yes or no), non-aspirin NSAID use (yes or no), and insulin treatment (yes or no). Model 2 included the adjustments for covariates in Model 1, plus several dietary factors, including cereal intake, fish intake, fruit intake, red meat intake, vegetable intake, vitamin supplements, mineral and fish oil supplements, and healthy diet score. For covariates with a missing rate > 5% (7.8% for optimal physical activity), we imputed mean values for continuous variables or created an additional category for categorical variables.

Furthermore, stratified analyses were performed to explore the potential modifying effects of APOE ε4 dosage, baseline cognitive function, and a range of covariates on the association of glucosamine use with risk of vascular dementia, Alzheimer’s disease.

We conducted all analysis using R version 4.0.1. In all statistical tests, the two-sided *P* value < 0.05 was considered to be statistically significant.

## Results

### Baseline characteristics of study participants

Of the 214,945 participants included, the mean age was 64.1 (SD: 2.9) years, 113,476 (52.8%) participants were female, and 52,893 (24.6%) participants were habitual glucosamine users.

The characteristics of the participants by using glucosamine supplement or not are showed in Table 1. Compared with glucosamine non-users, habitual glucosamine users were older, more likely to be female and non-smokers, and less likely to take anti-hypertensive, lowering cholesterol drugs, insulin treatment, and aspirin; had higher physical activity levels and lower levels of deprivation; consumed more alcohol and a healthier diet; and tended to take non-aspirin NSAID and supplements of vitamins, minerals, and fish oil. Moreover, glucosamine users had higher prevalence of family history of dementia and self-reported arthritis, and lower prevalence of diabetes, hypertension, and CVD (Table 1).

**Table 1:** Characteristics of the UK Biobank participants by glucosamine use

Characteristics	Total	Glucosamine non-users	Glucosamine users	*P* value
	*N* = 214,945	*N* = 162,052	*N* = 52,893	
Female, *n* (%)	113,476 (52.8)	80,613 (49.7)	32,863 (62.1)	< 0.001
Age, years	64.1 (2.9)	64.1 (2.9)	64.2 (2.8)	< 0.001
White race, *n* (%)	207,923 (97.1)	156,539 (97.0)	51,384 (97.4)	< 0.001
Household income, £				< 0.001
< 18,000	59,429 (27.8)	46,816 (29)	12,613 (23.9)	
18,000*–*30,999	56,958 (26.6)	41,803 (25.9)	15,155 (28.7)	
31,000*–*51,999	36,426 (17.0)	26,517 (16.5)	9909 (18.8)	
52,000*–*100,000	17,539 (8.2)	13,040 (8.1)	4499 (8.5)	
> 100 000	4182 (2.0)	3177 (2.0)	1005 (1.9)	
Townsend deprivation index	− 1.6 (3.0)	− 1.4 (3.0)	− 2 (2.7)	< 0.001
Body mass index, kg/m^2^	27.6 (4.6)	27.6 (4.6)	27.4 (4.5)	< 0.001
Optimal physical activity	118,718 (59.9)	87,525 (58.9)	31,193 (63)	< 0.001
Smoking status, *n* (%)				< 0.001
Never	106,920 (49.7)	79,245 (48.9)	27,675 (52.3)	
Former	89,309 (41.5)	67,034 (41.4)	22,275 (42.1)	
Current	17,655 (8.2)	14,924 (9.2)	2731 (5.2)	
Alcohol consumption,* n* (%)				< 0.001
Daily or almost daily	50,181 (23.3)	36,951 (22.8)	13,230 (25.0)	
3–4 times a week	47,371 (22)	34,796 (21.5)	12,575 (23.8)	
Once or twice a week	51,283 (23.9)	38,799 (23.9)	12,484 (23.6)	
1–3 times a month	21,258 (9.9)	16,118 (9.9)	5140 (9.7)	
Never or special occasions only	44,700 (20.8)	35,256 (21.8)	9444 (17.9)	
Healthy diet score	3.2 (1.4)	3.2 (1.4)	3.4 (1.4)	< 0.001
Family history of dementia, *n* (%)	32,271 (15.0)	23,823 (14.7)	8448 (16.0)	< 0.001
APOE ε4 dosage				0.290
0	151,036 (73.7)	113,815 (73.7)	37,221 (73.7)	
1	49,098 (23.9)	37,051 (24.0)	12,047 (23.8)	
2	4904 (2.4)	3651 (2.4)	1253 (2.5)	
Global cognitive function (z-score)	0.00 (0.73)	− 0.02 (0.74)	0.04 (0.67)	< 0.001
**Self-reported disease history, *n* (%)**				
Diabetes	15,076 (7.0)	12,749 (7.9)	2327 (4.4)	< 0.001
Hypertension	77,998 (36.3)	61,097 (37.7)	16,901 (32.0)	< 0.001
Arthritis	29,344 (13.7)	17,813 (11.0)	11,531 (21.8)	< 0.001
History of cardiovascular disease	27,694 (12.9)	22,995 (14.2)	4699 (8.9)	< 0.001
**Drug use, *n* (%)**				
Anti-hypertensive	68,098 (31.9)	53,976 (33.5)	14,122 (26.8)	< 0.001
Lowering cholesterol	59,983 (28.1)	47,578 (29.5)	12,405 (23.5)	< 0.001
Insulin treatment	2955 (1.4)	2541 (1.6)	414 (0.8)	< 0.001
Aspirin	45,315 (21.3)	35,662 (22.3)	9653 (18.4)	< 0.001
Non-aspirin NSAID	22,514 (10.6)	14,571 (9.1)	7943 (15.1)	< 0.001
**Supplement use, *n* (%)**				
Vitamin	36,008 (16.8)	22,521 (14)	13,487 (25.6)	< 0.001
Mineral and fish oil	96,999 (45.1)	59,005 (36.4)	37,994 (71.8)	< 0.001

Variables are presented as mean (SD) or *n* (%)

*NASID* non-steroidal anti-inflammatory drug

### Associations of glucosamine use with risk of incident cause-specific dementia

During a median follow-up duration of 12 years, 1039, 1774, and 122 participants developed incident vascular dementia, Alzheimer’s disease cases, and frontotemporal dementia, respectively.

Overall, habitual glucosamine use was significantly associated with a lower risk of incident vascular dementia (adjusted HR, 0.83; 95% CI, 0.70–0.97), but not significantly associated with the risk of incident Alzheimer’s disease (adjusted HR, 1.00; 95% CI, 0.89–1.11) and incident frontotemporal dementia (adjusted HR, 1.20; 95% CI, 0.78–1.85) (Table 2).

**Table 2:** Associations of glucosamine use with incident cause-specific dementia

Categories of dementia	Glucosamine non-users	Glucosamine users	*P* value
	*N* = 162,052	*N* = 52,893	
**Vascular dementia**			
No. of case	1039	229	-
Person-years	1,853,990	613,980	-
Age- and sex-adjusted model	1 [Reference]	0.68 (0.59, 0.78)	< 0.001
Model 1	1 [Reference]	0.82 (0.70, 0.96)	0.014
Model 2	1 [Reference]	0.83 (0.70, 0.97)	0.023
**Alzheimer’s disease**			
No. of case (%)	1774	581	
Person-years	1,852,132	613,119	
Age- and sex-adjusted model	1 [Reference]	0.97 (0.88, 1.07)	0.553
Model 1	1 [Reference]	1.02 (0.92, 1.14)	0.648
Model 2	1 [Reference]	1.00 (0.89, 1.11)	0.929
**Frontotemporal dementia**			
No. of case (%)	122	32	-
Person-years	1,855,706	614,363	-
Age- and sex-adjusted model	1 [Reference]	0.83 (0.56, 1.23)	0.348
Model 1	1 [Reference]	0.95 (0.63, 1.43)	0.804
Model 2	1 [Reference]	1.20 (0.78, 1.85)	0.401

Model 1: adjusted for age, sex (female or male), ethnicity (white, others), centers, body mass index, household income (< 18,000, 18,000–30,999, 31,000–51,999, 52,000–10,000, > 100,000 £/yr), Townsend deprivation index, smoking status (never, former, current), alcohol consumption (daily or almost daily, 3–4 times a week, once or twice a week, 1–3 times a month, never or special occasions only), optimal physical activity (yes or no), family history of dementia (yes or no), APOE ε4 dosage (0, 1, 2), self-reported diabetes (yes or no), self-reported hypertension (yes or no), self-reported arthritis (yes or no), history of cardiovascular disease (yes or no), antihypertensive drugs (yes or no), lipid treatment (yes or no), aspirin use (yes or no), non-aspirin NSAID use (yes or no), insulin treatment (yes or no)

Model 2: covariates in Model 1 plus cereal intake, fish intake, fruit intake, red meat intake, vegetable intake, vitamin supplements, mineral and other supplements, healthy diet score

Excluding participants with follow-up duration of less than 5 years (Supplementary table 1, Sensitivity analysis 1), or including baseline cognitive function scores in the adjustment (Supplementary table 1, Sensitivity analysis 2), did not materially alter the findings.

### Stratified analyses

**Fig. 1:**
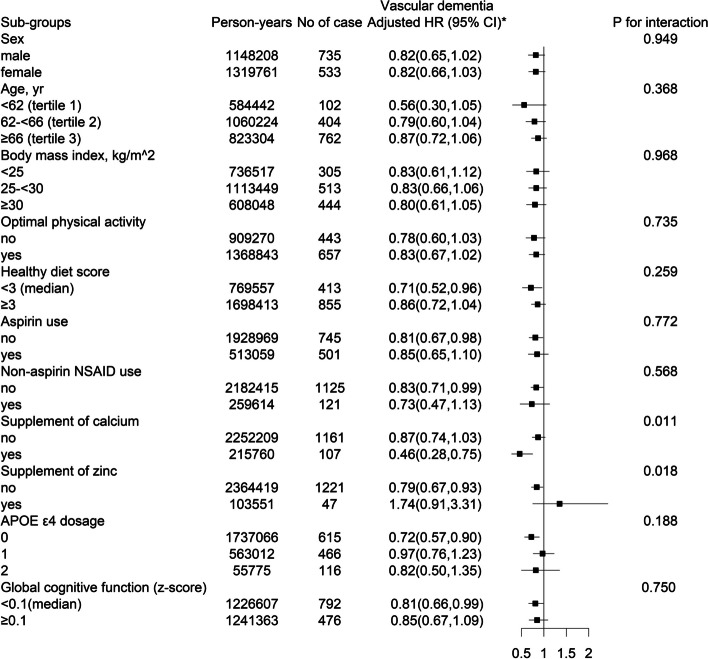
Stratified analyses of the association between glucosamine use and incident vascular dementia. ^*^Adjusted for age, sex (female or male), ethnicity (white, others), centers, body mass index, household income (< 18,000, 18,000–30,999, 31,000–51,999, 52,000–10,000, > 100,000 £/yr), Townsend deprivation index, smoking status (never, former, current), alcohol consumption (daily or almost daily, 3–4 times a week, once or twice a week, 1–3 times a month, never or special occasions only), optimal physical activity (yes or no), family history of dementia (yes or no), APOE ε4 dosage (0, 1, 2), self-reported diabetes (yes or no), self-reported hypertension (yes or no), self-reported arthritis (yes or no), history of cardiovascular disease (yes or no), antihypertensive drugs (yes or no), lipid treatment (yes or no), aspirin use (yes or no), non-aspirin NSAID use (yes or no), and insulin treatment (yes or no)

**Fig. 2:**
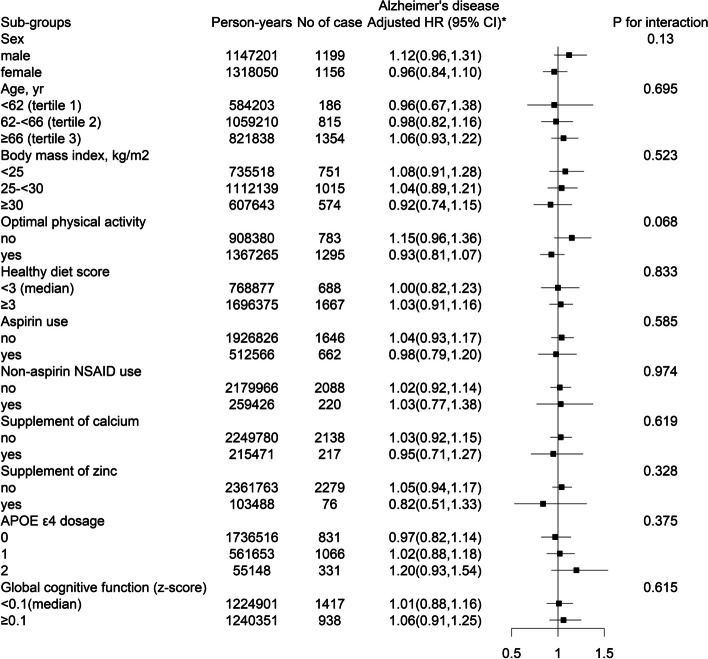
Stratified analyses of the association between glucosamine use and incident Alzheimer’s disease. ^*^Adjusted for age, sex (female or male), ethnicity (white, others), centers, body mass index, household income (< 18,000, 18,000–30,999, 31,000–51,999, 52,000–10,000, > 100,000 £/yr), Townsend deprivation index, smoking status (never, former, current), alcohol consumption (daily or almost daily, 3–4 times a week, once or twice a week, 1–3 times a month, never or special occasions only), optimal physical activity (yes or no), family history of dementia (yes or no), APOE ε4 dosage (0, 1, 2), self-reported diabetes (yes or no), self-reported hypertension (yes or no), self-reported arthritis (yes or no), history of cardiovascular disease (yes or no), antihypertensive drugs (yes or no), lipid treatment (yes or no), aspirin use (yes or no), non-aspirin NSAID use (yes or no), and insulin treatment (yes or no)

Stratified analyses were performed to assess the possible modifying factors on the association of glucosamine use with incident vascular dementia and incident Alzheimer’s disease (Figs. 1 and 2, and Supplementary Fig. 2).

A significantly stronger inverse association between glucosamine use and incident vascular dementia was found in participants with concomitant supplement of calcium (yes, adjusted HR, 0.46; 95% CI, 0.28–0.75; *vs.* no, adjusted HR, 0.87; 95% CI, 0.74–1.03; *P* for interaction = 0.011), and those without concomitant supplement of zinc (no, adjusted HR, 0.79; 95% CI, 0.67–0.93; *vs.* yes, adjusted HR, 1.74; 95% CI, 0.91–3.31; *P* for interaction = 0.018). Moreover, supplement of calcium and zinc did not significantly modify the association between glucosamine use and incident Alzheimer’s disease (both *P* for interactions ≥ 0.05) (Figs. 1 and 2, Supplementary Fig. 2).

As expected, APOE Ɛ4 dosage was significantly and positively associated with the risk of incident vascular dementia (2 *vs.* 0; adjusted HR: 5.81; 95%CI: 4.72, 7.15), and incident Alzheimer’s disease (2 *vs.* 0; adjusted HR: 12.05; 95%CI: 10.54, 13.77) (Supplementary Table 2). However, APOE ε4 dosage did not show significant modifying effects on the relationship of glucosamine use with incident vascular dementia (*P* for interaction = 0.188) and Alzheimer’s disease (*P* for interaction = 0.375). Other variables, including cognitive function, sex, age, BMI, physical activity, healthy diet score, self-reported arthritis, aspirin use, non-aspirin NSAID use, diabetes, hypertension, history of CVD, supplement of vitamin A, vitamin B, vitamin C, vitamin D, vitamin E, folate, selenium, iron, and fish oil, also did not significantly modify the associations of glucosamine use with risks of incident vascular dementia or Alzheimer’s disease associations (all *P* for interactions > 0.05) (Figs. 1 and 2, Supplementary Fig. 2).

## Discussion

Our study showed that in the older population, habitual supplement of glucosamine was significantly associated with a lower risk of incident vascular dementia, but not significantly associated with the risk of incident Alzheimer’s disease and frontotemporal dementia. APOE genetic variations and baseline cognitive function did not significantly modify this association.

A few studies [17, 18] have investigated the longitudinal association of habitual glucosamine use with incident dementia in the general population and have reported inconsistent results. Ai et al. [17] found that habitual supplementation of glucosamine was not associated with incident all-cause dementia in approximately 290,000 middle- to old-aged participants during a median follow-up of 9.1 years. However, Zheng et al. [18] showed that glucosamine use was associated with a lower risk of all-cause dementia, Alzheimer’s disease, and vascular dementia in approximately 490,000 middle- to old-aged participants over a median follow-up of 8.9 years. Of note, none of the previous studies have examined the association between glucosamine use and incident frontotemporal dementia. Moreover, although the high risk of dementia in older adults, no studies have specifically investigated the association between glucosamine use and incident dementia in the older population and fully explored the possible effect modifications on this association, particularly with regard to fish oil, minerals, and vitamins that are commonly used by older adults, and baseline cognitive function. Our current study, which has the longest follow-up duration compared to previous studies [17, 18], addresses the above knowledge gaps in time in the older population, by adjusting for a range of important confounding factors, and taking into account the modifying effect of baseline cognitive function and use of fish oil and a series of minerals and vitamins.

Our study provides some new insights. Firstly, in the older population, there was a significant inverse association of glucosamine use with incident vascular dementia. However, glucosamine use was not significantly associated with incident Alzheimer’s disease and frontotemporal dementia. Previous studies have found that glucosamine supplementation was associated with a reduced risk of CVD [5] and type 2 diabetes [13], both of which are associated with an increased risk of dementia, particularly vascular dementia [33, 34]. In addition, a previous randomized trial, including 38 subjects diagnosed with knee osteoarthritis, showed that glucosamine sulfate supplements could modulate the metabolic and immune activity of the gastrointestinal microbiota [35], thereby affecting the risk of neurodegenerative diseases [36]. All of these results suggest a possible beneficial effect of glucosamine on dementia.

The underlying mechanism for the benefit of glucosamine supplementation in vascular dementia is uncertain, but biologically plausible. First, glucosamine use may provide an anti-inflammation effect by interfering with nuclear factor-κB activity [9], therefore reducing angiogenesis and remodeling [37]. Second, glucosamine treatment could relieve inflammatory atherosclerosis at the femoral wall and aortic [38]. These beneficial effects of glucosamine supplement on vascular functions may play an important in the prevention of vascular dementia [39]. Of note, our data indicate a significant inverse association between glucosamine use and vascular dementia only, not Alzheimer’s disease and frontotemporal dementia, in the older population. It could be that different types of dementia have different mechanisms. For example, although vascular dementia is closely related to the biological processes of Alzheimer’s disease [40], vascular dementia is primarily due to vascular lesions [41], while the primary cause of Alzheimer’s disease is β amyloid deposition [42]. Glucosamine may have a stronger effect on vascular damage and thus a stronger association with vascular dementia. In addition, the hippocampus gradually shrinks and cortical density decreases in the elderly, which leads to a decrease in cell membrane receptors of brain cells, thus weakening the effect of glucosamine on Alzheimer’s disease and frontotemporal dementia.

Secondly, a stronger inverse association between glucosamine use and risk of vascular dementia among those with concomitant supplement of calcium, or those without concomitant supplement of zinc. A recent study in Sweden found that those who received calcium supplementation had a more than threefold risk of developing vascular dementia compared to those who did not [43]. Therefore, participants with concomitant use of calcium may have benefited more from the anti-inflammatory and vascular protective effects of glucosamine use. However, a Mendelian randomization study found that increased serum calcium levels were associated with a reduced risk of Alzheimer’s disease [44]. Therefore, more research is needed to confirm our findings and further examine the underlying mechanisms. At the same time, zinc is an essential mineral nutrient that is involved in many important biological processes, including maintaining insulin homeostasis, influencing inflammatory responses [45], and playing key structural roles in thousands of proteins [46]. A recent study has reported that zinc intake was inversely associated with the prevalence of low cognitive performance [47]. We speculate that zinc and glucosamine may share some of the mechanisms that are beneficial to dementia risk, thus diminishing the beneficial effects of glucosamine. However, it must be noted that interactions of calcium supplement or zinc supplement and glucosamine use on incident vascular dementia became non-significant after the Bonferroni correction. As such, due to the chance given multiple testing, our results are just hypotheses, and the clinical implication of these interactions needs to be evaluated with more studies.

Our study has several limitations. First, the UK Biobank did not collect more specific information on glucosamine use, including form, dose, frequency, duration, etc. Moreover, the information on supplement use in the UK Biobank was available at one time point; more frequent assessments could provide more accurate results. Second, although a broad range of covariates were included in the adjustments, possible confounding from other unknown or unmeasured factors could not be excluded. Third, the causation cannot be determined through the observational design in this analysis. Fourth, previous studies have reported different positive predictive values (PPV) for different types of dementia [31, 32], with vascular dementia and frontotemporal dementia in particular having relatively low PPV, which could lead to potential misclassification bias. In addition, dementia cases may have been underestimated due to the ascertainment based on ICD9/10 codes, leading to an underestimation of the true effect size. Fifth, the present study was conducted in UK older people with special socioeconomic status and overall health status, and 97% of the participants were white race [48]; whether the observed results can be extrapolated to other populations will need further investigation.

In summary, our study showed that habitual glucosamine use was significantly associated with a lower risk of incident vascular dementia in the older population, regardless of APOE genotypes and cognitive function. If further confirmed, habitual glucosamine use may act as a dietary supplement for primary prevention of vascular dementia in the elderly.

### Supplementary Information

**Additional file 1: Supplementary figure 1.** Flow chart of the study participants. **Supplementary figure 2.** Stratified analyses of the association between glucosamine use and incident vascular dementia (A), incident Alzheimer's disease (B) in other subgroups. **Supplementary table 1.** Sensitivity analysis for associations of glucosamine use with incident cause-specific dementia^*^. **Supplementary table 2.** Associations between APOE ε4 dosage and incident vascular dementia, incident Alzheimer's disease^*^.

## Data Availability

Data, analytic methods, and study materials that support the findings of this study will be available from the corresponding authors on request.

## Abbreviations

APOE: Apolipoprotein E
BMI: Body mass index
CVD: Cardiovascular disease
HR: Hazard ratio
NSAID: Non-steroidal anti-inflammatory drug
PPV: Positive predictive values
SD: Standard deviation
95%CI: 95% Confidence interval

## References

[1] Sibbritt D, Adams J, Lui CW, Broom A, Wardle J. Who uses glucosamine and why? A study of 266,848 Australians aged 45 years and older. PLoS One. 2012;7(7):e41540. 10.1371/journal.pone.0041540. 10.1371/journal.pone.0041540 PMC3408465 22859995

[2] Barnes PM, Bloom B, Nahin RL (2008). Complementary and alternative medicine use among adults and children: United States, 2007. Natl Health Stat Report.

[3] Ma H, Li X, Sun D, et al. Association of habitual glucosamine use with risk of cardiovascular disease: prospective study in UK Biobank. BMJ. 2019;365: l1628. 10.1136/bmj.l1628. 10.1136/bmj.l1628 PMC6515311 31088786

[4] Clegg DO, Reda DJ, Harris CL (2006). Glucosamine, chondroitin sulfate, and the two in combination for painful knee osteoarthritis. N Engl J Med.

[5] Hochberg MC, Martel-Pelletier J, Monfort J (2016). Combined chondroitin sulfate and glucosamine for painful knee osteoarthritis: a multicentre, randomised, double-blind, non-inferiority trial versus celecoxib. Ann Rheum Dis.

[6] Roman-Blas JA, Castañeda S, Sánchez-Pernaute O, Largo R, Herrero-Beaumont G; CS/GS Combined Therapy Study Group. Combined Treatment With Chondroitin Sulfate and Glucosamine Sulfate Shows No Superiority Over Placebo for Reduction of Joint Pain and Functional Impairment in Patients With Knee Osteoarthritis: A Six-Month Multicenter, Randomized, Double-Blind, Placebo-Controlled Clinical Trial. Arthritis Rheumatol. 2017; 69(1):77–85. 10.1002/art.39819. 10.1002/art.39819 27477804

[7] Runhaar J, Rozendaal RM, van Middelkoop M (2017). Subgroup analyses of the effectiveness of oral glucosamine for knee and hip osteoarthritis: a systematic review and individual patient data meta-analysis from the OA trial bank. Ann Rheum Dis.

[8] Beaudart C, Lengelé L, Leclercq V (2020). Symptomatic efficacy of pharmacological treatments for knee osteoarthritis: a systematic review and a network meta-analysis with a 6-month time horizon. Drugs.

[9] Largo R, Alvarez-Soria MA, Díez-Ortego I (2003). Glucosamine inhibits IL-1beta-induced NFkappaB activation in human osteoarthritic chondrocytes. Osteoarthritis Cartilage.

[10] Bascoul-Colombo C, Garaiova I, Plummer SF, Harwood JL, Caterson B, Hughes CE (2016). Glucosamine hydrochloride but not chondroitin sulfate prevents cartilage degradation and inflammation induced by interleukin-1α in bovine cartilage explants. Cartilage.

[11] Kantor ED, Lampe JW, Vaughan TL, Peters U, Rehm CD, White E (2012). Association between use of specialty dietary supplements and C-reactive protein concentrations. Am J Epidemiol.

[12] Hotamisligil GS (2006). Inflammation and metabolic disorders. Nature.

[13] Ma H, Li X, Zhou T (2020). Glucosamine use, inflammation, and genetic susceptibility, and incidence of type 2 diabetes: a prospective study in UK Biobank. Diabetes Care.

[14] Lee Y, Lee S, Park JW (2018). Hypoxia-induced neuroinflammation and learning-memory impairments in adult zebrafish are suppressed by glucosamine. Mol Neurobiol.

[15] Chou LY, Chao YM, Peng YC, Lin HC, Wu YL (2020). Glucosamine enhancement of BDNF expression and animal cognitive function. Molecules.

[16] Nevado-Holgado AJ, Kim CH, Winchester L, Gallacher J, Lovestone S. Commonly prescribed drugs associate with cognitive function: a cross-sectional study in UK Biobank. BMJ Open. 2016;6(11):e012177. 10.1136/bmjopen-2016-012177. 10.1136/bmjopen-2016-012177 PMC5168501 27903560

[17] Ai B, Chen L, Cai M, et al. No associations between glucosamine supplementation and dementia or Parkinson’s disease: Findings from a large prospective cohort study. J Gerontol A Biol Sci Med Sci. 2023;glad123. 10.1093/gerona/glad123. [Online ahead of print]. 10.1093/gerona/glad123 37158699

[18] Zheng J, Ni C, Zhang Y (2023). Association of regular glucosamine use with incident dementia: evidence from a longitudinal cohort and Mendelian randomization study. BMC Med.

[19] Kunkle BW, Grenier-Boley B, Sims R (2019). Genetic meta-analysis of diagnosed Alzheimer’s disease identifies new risk loci and implicates A β, tau, immunity and lipid processing. Nat Genet.

[20] Skillbäck T, Lautner R, Mattsson N (2018). Apolipoprotein E genotypes and longevity across dementia disorders. Alzheimers Dement.

[21] Sudlow C, Gallacher J, Allen N, et al. UK biobank: an open access resource for identifying the causes of a wide range of complex diseases of middle and old age. PLoS Med. 2015;12(3):e1001779. 10.1371/journal.pmed.1001779. 10.1371/journal.pmed.1001779 PMC4380465 25826379

[22] Davies G, Harris SE, Reynolds CA (2014). A genome-wide association study implicates the APOE locus in nonpathological cognitive ageing. Mol Psychiatry.

[23] Lyall DM, Ward J, Ritchie SJ (2016). Alzheimer disease genetic risk factor APOE e4 and cognitive abilities in 111,739 UK Biobank participants. Age Ageing.

[24] Malik R, Georgakis MK, Neitzel J (2021). Midlife vascular risk factors and risk of incident dementia: longitudinal cohort and Mendelian randomization analyses in the UK Biobank. Alzheimers Dement.

[25] Lloyd-Jones DM, Hong Y, Labarthe D (2010). Defining and setting national goals for cardiovascular health promotion and disease reduction: the American Heart Association’s strategic Impact Goal through 2020 and beyond. Circulation.

[26] Lichtenstein AH, Appel LJ, Brands M (2006). Diet and lifestyle recommendations revision 2006: a scientific statement from the American Heart Association Nutrition Committee. Circulation.

[27] Fawns-Ritchie C, Deary IJ. Reliability and validity of the UK Biobank cognitive tests. PLoS One. 2020;15(4): e231627. 10.1371/journal.pone.0231627. 10.1371/journal.pone.0231627 PMC7170235 32310977

[28] Cullen B, Smith DJ, Deary IJ, Evans JJ, Pell JP (2017). The 'cognitive footprint' of psychiatric and neurological conditions: cross-sectional study in the UK Biobank cohort. Acta Psychiatr Scand.

[29] Campbell T, Cullen B. Estimating the effect of physical activity on cognitive function within the UK Biobank cohort. Int J Epidemiol. 2023;dyad009. 10.1093/ije/dyad009. [Online ahead of print]. 10.1093/ije/dyad009 PMC10555922 36749099

[30] Li P, Gao L, Gao C (2022). Daytime sleep behaviors and cognitive performance in middle- to older-aged adults living with and without HIV infection. Nat Sci Sleep.

[31] Wilkinson T, Ly A, Schnier C (2018). Identifying dementia cases with routinely collected health data: a systematic review. Alzheimers Dement.

[32] Wilkinson T, Schnier C, Bush K (2019). Identifying dementia outcomes in UK Biobank: a validation study of primary care, hospital admissions and mortality data. Eur J Epidemiol.

[33] Xue M, Xu W, Ou YN, et al. Diabetes mellitus and risks of cognitive impairment and dementia: a systematic review and meta-analysis of 144 prospective studies. Ageing Res Rev. 2019; 55:100944. 10.1016/j.arr.2019.100944. 10.1016/j.arr.2019.100944 31430566

[34] Sattui SE, Rajan M, Lieber SB (2021). Association of cardiovascular disease and traditional cardiovascular risk factors with the incidence of dementia among patients with rheumatoid arthritis. Semin Arthritis Rheum.

[35] Coulson S, Butt H, Vecchio P, Gramotnev H, Vitetta L (2013). Green-lipped mussel extract (Perna canaliculus) and glucosamine sulphate in patients with knee osteoarthritis: therapeutic efficacy and effects on gastrointestinal microbiota profiles. Inflammopharmacology.

[36] Fang P, Kazmi SA, Jameson KG, Hsiao EY (2020). The microbiome as a modifier of neurodegenerative disease risk. Cell Host Microbe.

[37] Whiteford JR, De Rossi G, Woodfin A (2016). Mutually supportive mechanisms of inflammation and vascular remodeling. Int Rev Cell Mol Biol.

[38] Largo R, Martínez-Calatrava MJ, Sánchez-Pernaute O (2009). Effect of a high dose of glucosamine on systemic and tissue inflammation in an experimental model of atherosclerosis aggravated by chronic arthritis. Am J Physiol Heart Circ Physiol.

[39] Sachdev P, Kalaria R, O'Brien J (2014). Diagnostic criteria for vascular cognitive disorders: a VASCOG statement. Alzheimer Dis Assoc Disord.

[40] Chen Y, Liu Q, Liu J (2022). Revealing the modular similarities and differences among Alzheimer’s disease, vascular dementia, and Parkinson’s disease in genomic networks. Neuromolecular Med.

[41] Kalaria RN (2018). The pathology and pathophysiology of vascular dementia. Neuropharmacology.

[42] Jack CR, Bennett DA, Blennow K (2018). NIA-AA Research Framework: Toward a biological definition of Alzheimer’s disease. Alzheimers Dement.

[43] Kern J, Kern S, Blennow K (2016). Calcium supplementation and risk of dementia in women with cerebrovascular disease. Neurology.

[44] He Y, Zhang H, Wang T, Han Z, Ni QB, Wang K, Wang L, Zhang Y, Hu Y, Jin S, Sun BL, Liu G (2020). Impact of serum calcium levels on Alzheimer’s disease: a Mendelian randomization study. J Alzheimers Dis.

[45] Pompano LM, Boy E (2021). Effects of dose and duration of zinc interventions on risk factors for type 2 diabetes and cardiovascular disease: a systematic review and meta-analysis. Adv Nutr.

[46] Andreini C, Banci L, Bertini I, Rosato A (2006). Counting the zinc-proteins encoded in the human genome. J Proteome Res.

[47] Li S, Sun W, Zhang D (2019). Association of zinc, iron, copper, and selenium intakes with low cognitive performance in older adults: a cross-sectional study from National Health and Nutrition Examination Survey (NHANES). J Alzheimers Dis.

[48] Fry A, Littlejohns TJ, Sudlow C, Doherty N, Adamska L, Sprosen T, Collins R, Allen NE (2017). Comparison of sociodemographic and health-related characteristics of UK Biobank participants with those of the general population. Am J Epidemiol.

