# Supplementary material for: Habitual glucosamine use, APOE genotypes, and risk of incident cause-specific dementia in the older population
Source: Alzheimers Res Ther. 2023 Sep 9;15:152. doi: 10.1186/s13195-023-01295-6 (PMC10492372; doi:10.1186/s13195-023-01295-6)
Supplement: Supplementary file 1 — Additional file 1: Supplementary figure 1. Flow chart of the study participants. Supplementary figure 2. Stratified analyses of the association between glucosamine use and incident vascular dementia (A), incident Alzheimer's disease (B) in other subgroups. Supplementary table 1. Sensitivity analysis for associations of glucosamine use with incident cause-specific dementia*. Supplementary table 2. Associations between APOE ε4 dosage and incident vascular dementia, incident Alzheimer's disease*. [file 13195_2023_1295_MOESM1_ESM.doc]

502,461 participants

in the UK Biobank

Exclude: n=6,241

Withdraw: n=47;

Missing data on glucosamine use: n=6,194

496,220 participants

were included

Exclude: n=281,275

Report dementia at baseline: n=587;

With age <60 years: n= 280,688

214,945 participants

were finally analyzed

**Supplementary fig 1. Flow chart of the study participants**

**
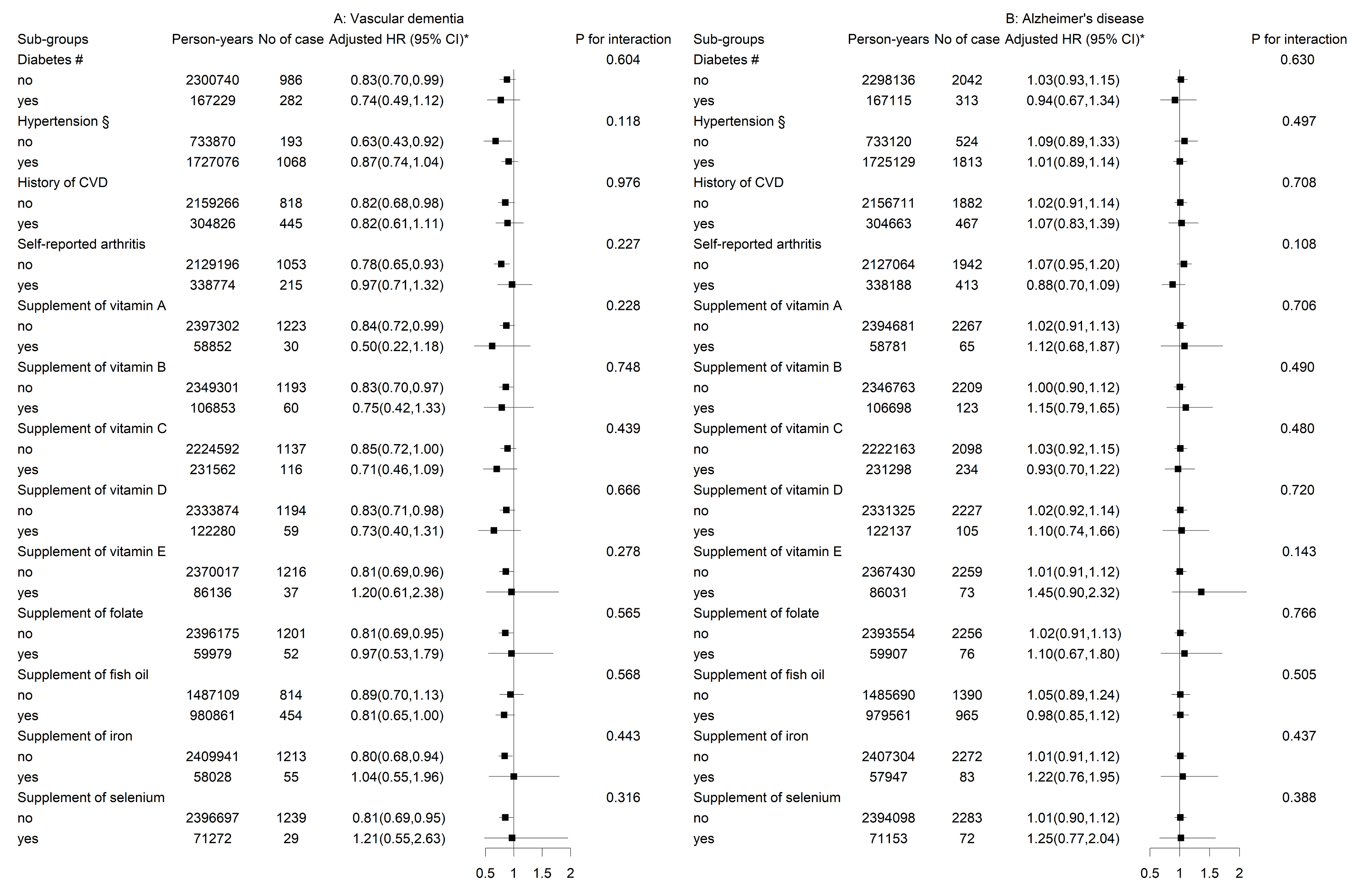
**

**Supplementary figure 2. Stratified analyses of the association between glucosamine use and incident vascular dementia (A), incident Alzheimer's disease (B) in other subgroups**

^*^Adjusted for age, sex (female or male), ethnicity (white, others), centers, body mass index, household income (<18000, 18000-30999, 31000-51999, 52000-10000, >100000 £/yr), Townsend deprivation index, smoking status (never, former, current), alcohol consumption (daily or almost daily, 3-4 times a week, once or twice a week, 1-3 times a month, never or special occasions only), optimal physical activity (yes or no), family history of dementia (yes or no), APOE ε4 dosage (0, 1, 2), self-reported diabetes (yes or no), self-reported hypertension (yes or no), self-reported arthritis (yes or no), history of cardiovascular disease (yes or no), antihypertensive drugs (yes or no), lipid treatment (yes or no), aspirin use (yes or no), non-aspirin NSAID use (yes or no), insulin treatment (yes or no). CVD, cardiovascular disease.

^#^ Diabetes was defined as self-reported diabetes, or use glucose-lowering drugs.

^§^ Hypertension was defined as self-reported hypertension, SBP/DBP ≥140/90 mmHg, or use antihypertensive drugs at baseline.

**Supplementary table 1. Sensitivity analysis for associations of glucosamine use with incident cause-specific dementia ^*^**

| Categories of dementia | Sensitivity analysis 1 | | Sensitivity analysis 2 | |
| --- | --- | --- | --- | --- |
|  | Adjusted HR (95%CI) | *P* value | Adjusted HR (95%CI) | *P* value |
| Vascular dementia | 0.85(0.72, 1.00) | 0.043 | 0.84(0.72, 0.98) | 0.028 |
| Alzheimer's disease | 1.07(0.96, 1.19) | 0.245 | 1.04(0.94, 1.15) | 0.472 |
| Frontotemporal dementia | 0.80(0.50, 1.27) | 0.346 | 0.98(0.65, 1.48) | 0.937 |

^*^ Adjusted for age, sex (female or male), ethnicity (white, others), centers, body mass index, household income (<18000, 18000-30999, 31000-51999, 52000-10000, >100000 £/yr), Townsend deprivation index, smoking status (never, former, current), alcohol consumption (daily or almost daily, 3-4 times a week, once or twice a week, 1-3 times a month, never or special occasions only), optimal physical activity (yes or no), family history of dementia (yes or no), APOE ε4 dosage (0, 1, 2), self-reported diabetes (yes or no), self-reported hypertension (yes or no), self-reported arthritis (yes or no), history of cardiovascular disease (yes or no), antihypertensive drugs (yes or no), lipid treatment (yes or no), aspirin use (yes or no), non-aspirin NSAID use (yes or no), insulin treatment (yes or no).

Sensitivity analysis 1: excluding participants with follow-up duration less than 5 years.

Sensitivity analysis 2: further including cognitive function scores in the adjustment.

**Supplementary table 2. Associations between APOE ε4 dosage and incident vascular dementia, incident Alzheimer's disease ^*^**

| APOE ε4 dosage | Vascular dementia | | Alzheimer's disease | |
| --- | --- | --- | --- | --- |
|  | Adjusted HR (95%CI) | *P* value | Adjusted HR (95%CI) | *P* value |
| 0 | Ref |  | Ref |  |
| 1 | 2.22(1.96, 2.52) | <0.001 | 3.82(3.47, 4.19) | <0.001 |
| 2 | 5.81(4.72, 7.15) | <0.001 | 12.05(10.54, 13.77) | <0.001 |

^*^ Adjusted for age, sex (female or male), ethnicity (white, others), centers, body mass index, household income (<18000, 18000-30999, 31000-51999, 52000-10000, >100000 £/yr), Townsend deprivation index, smoking status (never, former, current), alcohol consumption (daily or almost daily, 3-4 times a week, once or twice a week, 1-3 times a month, never or special occasions only), optimal physical activity (yes or no), family history of dementia (yes or no), self-reported diabetes (yes or no), self-reported hypertension (yes or no), self-reported arthritis (yes or no), history of cardiovascular disease (yes or no), antihypertensive drugs (yes or no), lipid treatment (yes or no), aspirin use (yes or no), non-aspirin NSAID use (yes or no), insulin treatment (yes or no).
